# A 6-Year-Old Child with Severe Ebola Virus Disease: Laboratory-Guided Clinical Care in an Ebola Treatment Center in Guinea

**DOI:** 10.1371/journal.pntd.0004393

**Published:** 2016-03-24

**Authors:** Romain Palich, Jean-Luc Gala, Frédéric Petitjean, Susan Shepherd, Olivier Peyrouset, Bing M. Abdoul, Moumouni Kinda, Christine Danel, Augustin Augier, Xavier Anglaret, Denis Malvy, Nicole Blackwell, ALIMA N’zérékoré Ebola Treatment Center medical group

**Affiliations:** 1 Alliance for International Medical Action, Dakar, Senegal; 2 Center for Applied Molecular Technologies, Louvain Catholic University, Brussels, Belgium; 3 Inserm, Centre 1219, Bordeaux, France; 4 Department of Tropical Medicine and Clinical International Health, Division of Infectious and Tropical Diseases, University Hospital of Bordeaux, Bordeaux, France; 5 Department of Critical Care, Division of Anesthesiology and Critical Care, University of Queensland, Brisbane, Australia; CDC, UNITED STATES

## Context

An unprecedented epidemic of Ebola virus disease has ravaged West Africa since December, 2013 [1].

The French nongovernmental organization Alliance for International Medical Action opened the N’zérékoré Ebola treatment center (ETC) on December 2nd, 2014. This center includes a laboratory managed by the Belgian organization B-Fast/B-Life. Between December 2nd, 2014, and February 7th, 2015, 130 patients were admitted, of whom 76 were confirmed to have Ebola virus disease (EVD), which was fatal in 60.8%.

Since December 26th, 2014, the N’zérékoré ETC has been a clinical trial site, participating in a multicenter study to evaluate the ability of a 10-day course of the oral antiviral agent favipiravir to reduce mortality in Ebola-infected adults and children aged over 1 year [2].

## Case Presentation

Our 6-year-old patient was likely infected by his mother, who was admitted to the center with EVD on December 19th, 2014. She died 2 days later. The patient’s father was admitted to the center on December 31st and tested positive. He cleared Ebola virus (EBOV) as of January 5th but remained in the ward as long as his child was hospitalized. The patient’s aunt has given consent to the publication of this case report.

## Clinical and Biological Status at Baseline, Initial Management

**Fig 1:**
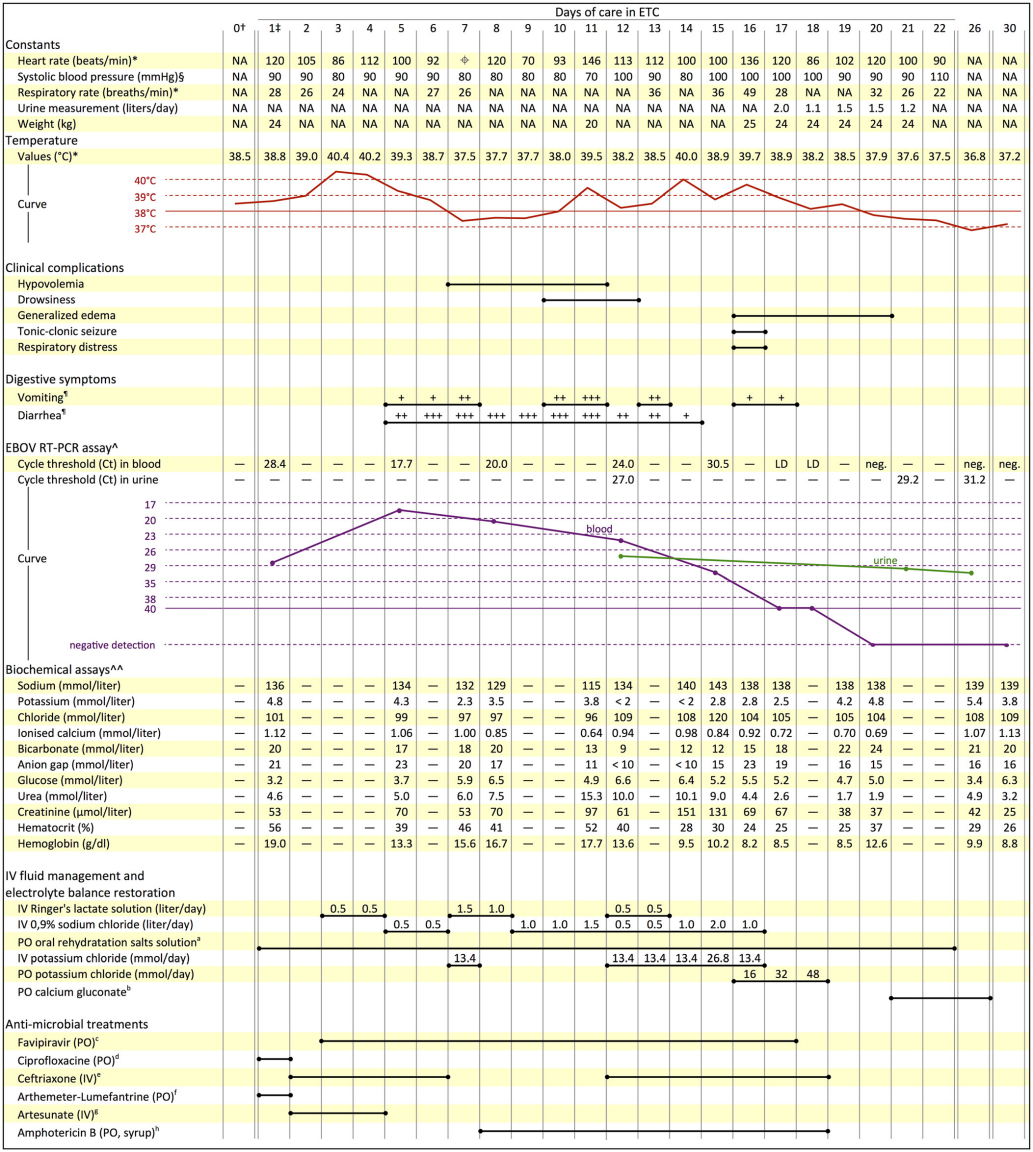
Clinical and biological outcomes and main treatments administered during care in the Ebola treatment center. ND: not available. LD: limit of detection. †December 25th, 2014. ‡December 26th, 2014. *Highest measurement of the day. §Lowest measurement of the day. ⌖Pulse was not perceived because of hemodynamic disorder. ¶Gastrointestinal losses were quantified at each visit of the medical team (four times per day): severe (+++), moderate (++), or mild (+). Severe losses: more than eight liquid stools per day (or more than four bouts of vomiting daily). Moderate losses: more than four liquid stools (or more than two bouts of vomiting per day). Mild losses: at least one liquid stool per day (or at least one bout of vomiting per day). ^Biomolecular tests employed RealStar Filovirus RT-PCR Kit 1.0 (Altona Diagnostics GmbH, Hamburg, Germany). ^^Biochemical assays employed i-STAT CHEM8+ cartridges (Abbott Point of Care Inc., Princeton, New Jersey, USA). ^a^Between 0.5 and 1.0 liter per day, at will. ^b^3.5 mmol per day. ^c^Loading dose of 3,000 mg on the first day (H0: 1,200 mg, H8: 1,200 mg, H16: 600mg) then 1,200 mg (600 mg twice a day) on the following days. ^d^250 mg twice per day. ^e^1 gram once a day. ^f^40 mg/240 mg twice a day. ^g^120 mg (H0 60 mg, H12 60 mg) on the first day, then 60 mg per day on the following days. ^h^500 mg twice a day.

The child was admitted to the ETC on December 26th, 2014, with isolated fever (38.8°C) that lasted for less than 24 hours. Cardiorespiratory parameters were stable. EBOV reverse-transcriptase polymerase chain reaction (RT-PCR) assay was positive (cycle threshold: 28.4), and blood chemistry values were as shown in Fig 1. Oral ciprofloxacin and artemether/lumefantrine combination were administered, as per the center protocol. On day 2, the patient showed lethargy, with decreased oral intake. Intravenous treatment was commenced, and antibiotic and antimalarial medications were changed to ceftriaxone and artesunate. The investigational drug favipiravir was initiated 36 hours post-admission (Fig 1), with a good feasibility of use. Comprehensive symptomatic treatment was initiated and included paracetamol, ondansetron, and loperamide.

## Subsequent Clinical Course and Biological Features

Clinical and biological parameters and treatments are detailed in Fig 1.

The patient experienced profuse diarrhea and vomiting on days 5–14, accompanied by hypotension, tachycardia, and drowsiness. He was treated for hypovolemia with intravenous fluid. Over these 10 days, 1 to 2 liters of 0.9% sodium chloride or Ringer’s lactate solution were administered daily to restore cardiac output and correct renal function. Hypokalemia, hypocalcemia, and metabolic acidosis were corrected by intravenous and oral treatments.

The patient was severely hyponatremic on day 11 (sodium 115 mmol per liter), with an 18% decrease in weight since admission but preserved consciousness.

Nutritional support included BP100 biscuits, providing potassium and contributing to oral electrolyte supplementation. Urine output measurement was not possible until day 17, when the patient’s father assisted with monitoring.

On day 8, the patient developed oral candidiasis with dysphagia, resolving after 10 days of oral amphotericin B.

The patient became afebrile on day 7, but fever recurred on day 11, with onset of abdominal pain and diffuse guarding. Ceftriaxone treatment was resumed from day 12 to day 18. His condition deteriorated on day 16. He developed generalized edema and had two tonic-clonic seizures; his temperature was 39.7°C. The seizures spontaneously resolved after 2 minutes, without use of benzodiazepines. He was tachypnoeic at 49 breaths per minute, without flaring of the nostrils, grunting, or intercostal muscle recession. Lying flat on his side did not aggravate his dyspnea. Oxygen saturation measurement was unavailable. Neurologic examination showed no focal abnormality or nuchal rigidity.

A urine dipstick test was negative for protein. A rapid test for *Plasmodium falciparum* (SD-Bioline, Standard Diagnostics Inc., Republic of Korea) was negative. Blood tests showed hypokalemia, metabolic acidosis, and anemia. Intravenous rehydration was ceased, diuretics were not used, and oral potassium supplementation was prescribed as indicated in Fig 1. Respiratory symptoms improved within hours and edema resolved over 7 days. No further seizures occurred.

No significant bleeding occurred. The patient experienced slight rectal bleeding on days 7 and 8 and venipuncture site bleeding on day 11. Other symptoms were nonspecific, including prolonged listlessness, headache, cough, conjunctivitis, myalgia, and arthralgia.

## EBOV RT-PCR Assays and Favipiravir Use

Ebola virus infection was confirmed by semiquantitative RT-PCR. Hence, according to the manufacturer’s instructions, results are expressed as the “cycle threshold” (Ct), corresponding to the number of cycles required to cross the threshold positivity value, which is thus inversely correlated with viral load. Between December 2nd, 2014, and January 16th, 2015, the lowest Ct among all patients was 15, with a Ct of 40 corresponding to the limit of viral detection.

Initial Ct was 28.4, reaching a nadir of 17.7 on day 5. The subsequent increase in Ct, reflecting decrease in viral load, was slow. The threshold of detection was reached on day 17, and EBOV RT-PCR tests were negative from day 20 onwards. Although the favipiravir trial protocol stipulates a 10-day treatment course, it was decided, in agreement with the study investigators, to continue the drug until the Ct corresponded to the upper limit of virus detection (day 14 of treatment, which was day 17 of disease).

## Cure and Discharge

Cure was declared on day 22 after 3 afebrile days, and the patient was discharged. Neurologic and respiratory examinations were normal. Laboratory results normalized on day 30 (Fig 1).

## Case Discussion

We report the survival of a 6-year old boy with complicated EVD. He survived despite his high viral load at admission [3]. However, age as a poor prognostic factor is controversial [4,5]. The case fatality rate seems very high in children under 5 years of age, but school children and adolescents seem to survive more. Our patient may be at this “age threshold.”

Our patient clearly illustrates the clinical course of severe EVD. Indeed, it seems difficult to refer the observed complications to electrolyte disturbances or direct pathogenesis of EBOV [6].

Viral load was monitored throughout the course of illness. Viral decay kinetics were slower than reported in other patients with EVD [3]. Instead of administering favipiravir for 10 days, as stipulated by the trial protocol [2], it was decided to pursue this therapy until the cycle threshold corresponded to the upper limit of virus detection, assuming that this could favor survival. This limit was reached on day 14 of treatment.

Restoring appropriate fluid and electrolyte balance is one of the keys to survival. We have shown that even in extreme conditions, individualized care with close laboratory monitoring can be provided in a West African ETC.

**Key Learning Points**

- Enhanced supportive care in young children with Ebola virus disease is crucial for favorable outcomes.
- Individual laboratory analyses are essential to target electrolyte replacement and meticulous maintenance of volume status and can be achieved with portable laboratories.
- Clinical trials should take into account appropriate dose usage in children with Ebola virus disease.
